# Habitual coffee consumption and office, home, and ambulatory blood pressure: results of a 10-year prospective study

**DOI:** 10.1097/HJH.0000000000003709

**Published:** 2024-04-22

**Authors:** Fosca Quarti Trevano, Sara Vela-Bernal, Rita Facchetti, Cesare Cuspidi, Giuseppe Mancia, Guido Grassi

**Affiliations:** a Clinica Medica, Department of Medicine and Surgery, University Milano-Bicocca, Milan, Italy; b Cardiometabolic Risk and Diabetes Research Group, INCLIVA Biomedical Research Institute and Internal Medicine Hospital Clinico de Valencia, Valencia, Spain; c University Milano-Bicocca, Milan, Italy

**Keywords:** ambulatory blood pressure, blood pressure variability, clinic blood pressure, coffee, gender, home blood pressure, new hypertension

## Abstract

**Objectives:**

Heterogeneous are the results of the published studies aimed at determining the long-term effects of habitual coffee consumption on blood pressure (BP). Specifically, no data are available on the longitudinal association between habitual coffee consumption and office, home and 24 h BP profile and variability.

**Methods:**

In 1408 subjects recruited in the Pressioni Arteriose Monitorate E Loro Associazioni (PAMELA) study, followed for a 10 year follow-up period and classified as coffee consumers and nonconsumers (self-reporting), we prospectically investigated the association between habitual coffee consumption and office, home and 24-h ambulatory BP; 24-h BP variability; and development of a new hypertensive state. Data were also analysed according to gender.

**Results:**

When data were adjusted for confounders habitual coffee nonconsumers and consumers displayed similar long-term BP changes during the follow-up in office, home, and ambulatory BP. No difference was found between heavy and moderate coffee consumers. Furthermore, also new-onset hypertension and patterns of BP variability were superimposable in coffee nonconsumers and consumers, independently on confounders including gender, number, and characteristics of the antihypertensive drug treatment.

**Conclusion:**

The present study, which is the first longitudinal investigation never performed examining in a prospective fashion the long-term (10 year) effects of coffee consumption on office, home, and ambulatory BP, provides conclusive evidence that habitual coffee consumption is associated with neutral effects on in-office and out-of-office BP values and related variabilities. This is the also the case for the new-onset hypertensive state.

## INTRODUCTION

Despite the consistent number of original investigations and meta-analyses published during the past two decades [1–16], whether and to what extent habitual coffee consumption is associated with increased, unchanged, or reduced blood pressure (BP) values remains controversial. A number of intrinsic limitations of the published studies are behind these uncertain information, including the fact that with only very few exceptions [6,16], the available data have been collected in the context of investigations performed with a cross-sectional design, precluding any prospective information to be gathered, and all the above mentioned evaluations (including the few prospective studies) have been performed taking into account only clinic BP values, thus preventing to obtain data on BP values outside the clinical environment. A recent cross-sectional study by our group, however, overcame the latter limitation, analysing office as well as home and 24-h ambulatory BP data [17].

The present study was designed to overcome the first limitation, providing prospective information on the association between habitual coffee consumption, BP, and the development of a new hypertensive state, based on values collected via office, home, and ambulatory measurements. An additional relevant evaluation was represented by the assessment of the impact of habitual coffee consumption on 24-h BP variability, that is, a parameter, which is closely related to subclinical organ damage and cardiovascular events independently on absolute BP load [18]. The data were analysed in the whole group of subjects and in two subgroups defined according to gender. The unique feature of the study, which examines data collected in the context of the Pressioni Arteriose Monitorate E Loro Associazioni (PAMELA) research project, is represented by its follow-up duration (10 years) [19], which is the longest carried out in population studies based on both in-office and out-of-office BP measurements aimed at assessing the association between habitual coffee consumption and BP.

## METHODS

### Study population

The PAMELA study was performed in 3200 subjects, representative of the population of Monza (a town near Milan, Italy) stratified according to sex, age (decades), and other characteristics from 25 to 74 years of age [20]. At the initial evaluation, carried out between 1990 and 1993, participation rate was 64% and thus data were available in 2051 subjects. The demographic and clinical characteristics of participants and nonparticipants, as assessed by phone interviews, were similar. As described in detail elsewhere, after an informed consent, participants were invited to the outpatient clinic of the S. Gerardo Hospital of Monza in the morning of a working day (Monday to Friday), following an overnight fast and abstinence from alcohol and smoking since the previous day [20]. The experimental protocol of the PAMELA study and the process for obtaining informed consent were approved by the Institutional Review Committee of the University Milano-Bicocca.

### Measurements

Data collected include medical history and physical examination on cardiovascular risk factors (overweight, cigarette smoking habit, alcohol intake, diabetes mellitus) weight, height, abdominal circumference, standard blood examinations, office, home, and 24-h ambulatory BP [20]. Height and weight were obtained to calculate BMI. In the questionnaire administered to the subjects, a specific question was related to dietary modifications during follow-up. Laboratory analyses included plasma glucose, total and high-density lipoprotein (HDL) plasma cholesterol and plasma triglycerides [20]. Glomerular filtration rate was estimated by the Chronic Kidney Disease EPIdemiology (CKD-EPI) equation. Low-density lipoprotein (LDL) cholesterol was estimated according to the Friedewald equation. Office BP was measured with the subject in the sitting position, using a mercury sphygmomanometer and taking the first and fifth Korotkoff sounds to identify systolic and diastolic values, respectively [20]. To assess ambulatory BP, subjects were fitted with an ambulatory BP monitoring device (Spacelabs 90207, Issaquah, Washington, USA) set to obtain automated oscillometric BP and heart rate readings every 20 min over 24 h [20]. Subjects were asked to pursue their normal activities during the monitoring period, holding the arm still at time of the BP readings, going to bed not later than 11.00 p.m. and arising not before 7.00 a.m. Subjects were also asked to self-measure BP at home, with a validated semiautomatic oscillometric device (Model HP 5331, Philips, Amsterdam, The Netherlands), with a cuff size appropriate to each individual's arm circumference, at 7.00 a.m. and 7.00 p.m., using the arm contralateral to the one used for ambulatory monitoring [20]. Participants were again contacted from 2001 to 2003, that is, after a mean time interval of 10.7 ± 0.61 years, and those willing to be re-examined were asked to attend the San Gerardo University Hospital for a second set of data collection, according to the same procedures used for the first set of data collection [19]. In each subject, the three office and two home BP measurements as well as the corresponding heart rate values were separately averaged. After editing for artifacts, all ambulatory BP recordings were analysed to obtain 24 h, average SBP and DBP, as well as mean BP, and heart rate, the overall 24-h variability being taken as the corresponding standard deviation around the average [18]. New-onset hypertension detected after 10 year follow-up was defined as an elevation in office, home or 24 h BP (SBP and/or DBP ≥140/90 mmHg for office, ≥135/85 mmHg for home and ≥130/80 mmHg for ambulatory BP) or new antihypertensive drug treatment [19].

Information on coffee consumption were obtained via the administration of a questionnaire in which the number of caffeine-containing coffees drunk per day was considered (from 0 to 1–2 and ≥3 cups of coffee). Decaffeinated coffee, tea, and other caffeinated drinks were not examined in the present study. The caffeine content per cup of ‘expresso’ Italian coffee, which was the most frequently consumed type of coffee by the PAMELA participants, averages 100 mg.

### Protocol and data analysis

Subjects were subdivided into three groups according to the number of coffee cups drunk per day (0, 1–2, and ≥ 3 cups of coffee). Measurements were carried out at the study entry and 10 years later. Only subjects who maintained during the follow-up the same category of coffee consumption (coffee consumers/nonconsumers) underwent the present analysis. Data related to subjects’ characteristics were analysed by descriptive statistics. Calculations included means and standard deviations of continuous variables as well as numbers and percentages of categorical variables. ANOVA and chi-square test were applied to compare groups with different levels of coffee assumption. Bonferroni correction was used when two groups were compared. Repeated measures mixed models were used to compare BP values during time among groups with different levels of coffee assumption. Because age, BMI, smoking habit, HDL cholesterol, estimated glomerular filtration rate values, and antihypertensive drug treatment were different among coffee consumer groups, models were also adjusted for these four variables. Same analysis was separately performed in male and female individuals. In the case of antihypertensive pharmacological treatment, additional analysis was carried out examining in the different coffee consumer groups the number and type antihypertensive drugs used at baseline and during follow-up. New-onset office, or home or 24-h hypertension was calculated in subjects with normal level of blood pressure and without the use of antihypertensive treatment at baseline. Odd ratios (ORs) were calculated by logistic regression models and they were adjusted for age, sex, and relative mean blood pressure. Cups of coffee equal to zero was used as reference group. A *P* value less than 0.05 was considered statistically significant. Statistical analysis was performed by SAS System (version 9.4; SAS Institute Inc, Cary, North Carolina, USA).

## RESULTS

From the original sample of 2051 subjects, 1408 attended both the two surveys and reported information about coffee consumption. Of them 1176 remained stable at the two surveys regarding their belonging to the category of coffee nonconsumers (*n* = 102) or consumers (*n* = 1074). Table 1 reports demographic and clinical characteristics of these subjects, subdivided in three groups according to the different levels of daily coffee consumption detected at the study entry. Habitual coffee consumers three or more cups per day were younger than consumers one to two cups and displayed slightly but significantly greater BMI values. They were also more frequent cigarette smokers. Plasma glucose, triglycerides, total cholesterol, LDL-cholesterol values were similar in the three groups of subjects, whereas HDL-cholesterol was significantly lower and estimated glomerular filtration rate significantly higher in coffee consumers who consume three or more cups per day. At the study entry, antihypertensive drug treatment was significantly less represented in coffee consumers who consume three or more cups per day than in the other two groups. The modifications in antihypertensive drug treatment, alcohol drinking habit, smoking, and BMI at the 10-year follow-up in nonconsumers and consumers are shown in Tables 2 and 3. In all the three groups, antihypertensive treatment and use of different classes of drugs significantly and quite homogeneously increased and this was the case also for BMI, whereas the percentage of smokers and alcohol drinkers remained substantially unmodified. No substantial modification of the dietary habit was reported in about 85% of participants during the follow-up.

**TABLE 1:** Demographic and clinical variables in the study population, subdivided in three groups according to chronic coffee consumption reported at the study entry

	Cups of coffee per day (number)		
Variable	0	1–2	≥3
Subjects (number)	102	471	603
Age (years)	49.4 ± 14.5	50.7 ± 13.6	46.9 ± 11.8^a^
Male (%)	51	48	55.2
BMI (kg/m^2^)	23.8 ± 3.8	25.3 ± 4.2^b^	25.4 ± 3.8^b^
Physically active (%)	34.3	28	29
Alcohol drinkers (%)	39.2	55.2^b^	49.8
Smokers (%)	14.7	17.8	38.3^b^^a^
Serum glucose (mg/dl)	89.9 ± 12.8	90.6 ± 22.6	89.4 ± 19.4
Total cholesterol (mg/dl)	222.1 ± 37.9	221.5 ± 42.9	224 ± 42.3
HDL cholesterol (mg/dl)	58.1 ± 16.6	57.4 ± 16.3	55.0 ± 15.5^b^
LDL cholesterol (mg/dl)	142.5 ± 37.0	142.4 ± 39.8	146.4 ± 38.5
Triglycerides (mg/dl)	107.6 ± 66.9	108.2 ± 63.5	113.6 ± 69.7
eGFR (ml/min/1.72 m^2^)	88.6 ± 14.4	87 ± 15.4	91.1 ± 14.4^a^
Anti-HT treatment (%)	17.6	18.9	11.9^a^

Data are shown as means ± standard deviation or percentages. Anti-HT, antihypertensive drug; eGFR, estimated glomerular filtration rate; HDL, high density lipoprotein; LDL, low density lipoprotein; NS, not significant.

a *P* < 0.05 vs. one to two cups of coffee per day.

b *P* < 0.05 vs. 0 cups of coffee per day.

**TABLE 2:** Modifications of different variables during the 10 year follow-up in the three groups of the study population of Table 1

	Cups of coffee per day (number)					
	0		1–2		≥3	
Variable	Entry	10-year	Entry	10-year	Entry	10-year
Anti-HT treatment (%)	17.6	32.4^∗^	18.9	39.3^∗^	11.9^∗^	29.2^∗^
Alcohol drinkers (%)	39.2	37.3	55.2	58.4	49.8	49.8
Smokers (%)	14.7	14.7	17.8	19.3	38.3	37.0
BMI (kg/m^2^)	23.8 ± 3.8	25.1 ± 3.9^∗^	25.3 ± 4.2	26.6 ± 4.4^∗^	25.4 ± 3.8	27 ± 4.4^∗^
HDL (mg/dl)	58.1 ± 16.6	61.8 ± 15^∗^	57.4 ± 16	61.9 ± 15^∗^	55.0 ± 15	59.0 ± 15
eGFR (ml/min/1.72 m^2^)	88.6 ± 14.4	83.3 ± 18.1^∗^	87 ± 15.4	80.1 ± 18.2^∗^	91.1 ± 14.4	84.9 ± 17.8^∗^

Data are shown as percentage (%) values and as means ± standard deviation. Asterisks (^∗^*P* < 0.05) refer to the statistical significance between data collected after 10 year follow-up vs. study entry. Anti-HT, antihypertensive drug; eGFR, estimated glomerular filtration rate; HDL, high density lipoprotein.

**TABLE 3:** Modifications of antihypertensive drug treatment during the 10-year follow-up in in the three groups of the study population of Table 1

	Cups of coffee per day (number)					
	0		1–2		≥3	
Variable	Entry	10-year	Entry	10-year	Entry	10-year
Number of anti-HT treatment [n (%)]							
0	84 (83.2%)	69 (68.3%)^∗^	382 (81.6%)	286 (60.9%)^∗^	531 (88.7%)	427 (70.9%)^∗^
1	9 (8.9%)	15 (14.9%)	45 (9.6%)	90 (19.2%)	43 (7.2%)	79 (13.1%)
2	8 (7.9%)	10 (9.9%)	31 (6.6%)	68 (14.5%)	22 (3.7%)	70 (11.6%)
≥3	0 (0%)	7 (6.9%)	10 (2.1%)	26 (5.5%)	3 (0.5%)	26 (4.3%)
Drug classes [*n* (%)]							
Beta blockers	7 (6.9%)	11 (10.9%)	31 (6.6%)	62 (13.2%)^∗^	27 (4.5%)	61 (10.1%)^∗^
Calcium blockers	2 (2%)	10 (9.9%)^∗^	24 (5.1%)	50 (10.6%)^∗^	7 (1.2%)	43 (7.1%)^∗^
Diuretics	8 (7.9%)	14 (13.9%)	50 (10.7%)	73 (15.5%)^∗^	35 (5.8%)	73 (12.1%)^∗^
ACEI/sartans	6 (5.9%)	15 (14.9%)^∗^	22 (4.7%)	83 (17.7%)^∗^	21 (3.5%)	85 (14.1%)^∗^
Vasodilators	2 (2%)	1 (1%)	4 (0.9%)	8 (1.7%)	3 (0.5%)	13 (2.2%)^∗^

Data are shown as absolute numbers (*n*) and percentage (%) values. Asterisks (^∗^*P* < 0.05) refer to the statistical significance between data collected after 10 year follow-up vs. study entry. ACEI, angiotensin converting enzyme inhibitors; Anti-HT, antihypertensive drug.

**FIGURE 1:**
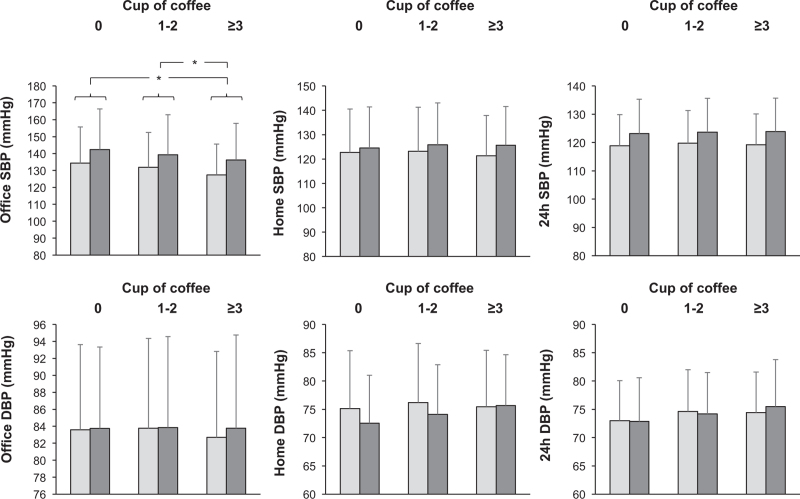
Office, home and 24 h ambulatory SBP (upper panels) and DBP (lower panels) blood pressure in coffee nonconsumers (0) and in coffee consumers one to two or at least three cups per day. Data are shown as means ± standard deviation at the study entry (grey columns) and after 10 year follow-up (black columns). Asterisks refer to the statistical significance (^∗^*P* < 0.05) between groups. Data are adjusted for age, BMI, smoking habit, antihypertensive drug treatment, HDL cholesterol, and estimated glomerular filtration rate values.

Data related to office, home, and 24 h BP values adjusted for confounders (age, BMI, smoking, and antihypertensive treatment) in nonconsumers and consumers at the study entry and after 10-year follow-up are shown in Fig. 1. In nonconsumers and consumers, office SBP values (left upper panel) were greater at the 10-year follow-up while DBP (left lower panel) remained substantially unchanged in all groups. Both at baseline and at the 10 year evaluation, office SBP values were significantly lower in consumers three cups of coffee per day as compared with the other two groups. In contrast, home and 24 h SBP and DBP values seen at the study entry and at the 10-year follow-up were not significantly different in the three groups of subjects (Fig. 1, middle and right panels). Office and out-of-office heart rate values were similar in coffee nonconsumers and consumers both at the study entry and at the 10-year follow-up (data not shown).

**FIGURE 2:**
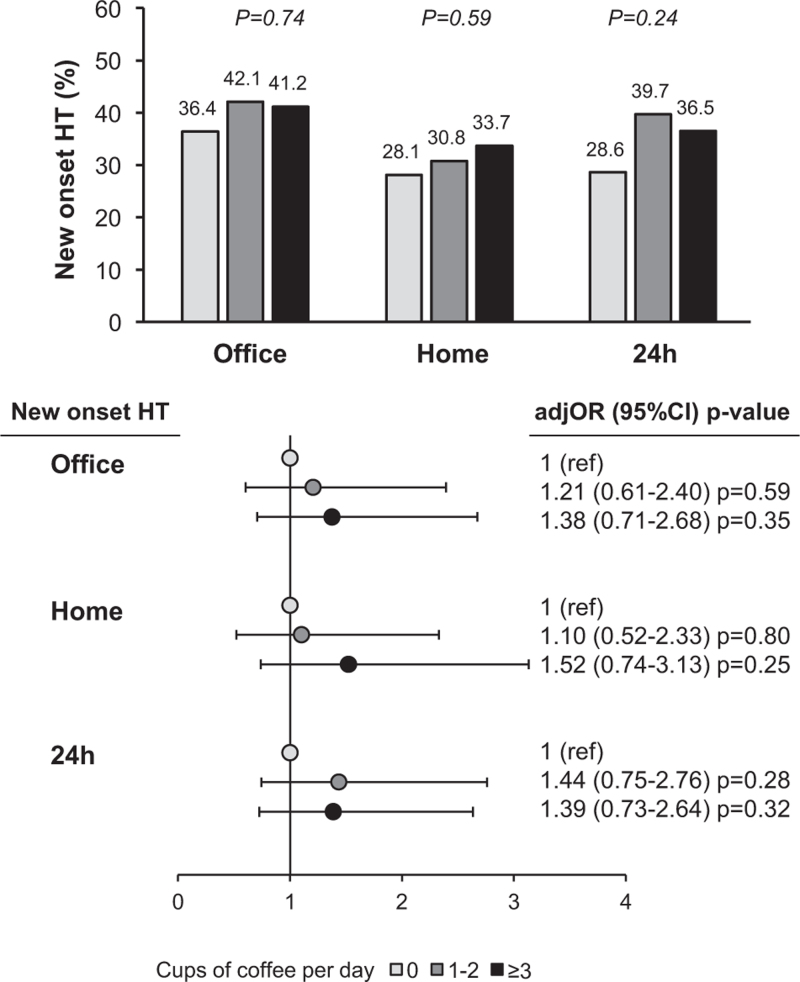
New onset of office, home, and 24 h hypertension at the 10 year follow-up. Data refer to normotensive subject at baseline (*n* = 421 for office, *n* = 422 for home and *n* = 436 for 24 h BP measurements). Upper panel reports percentage of new-onset hypertension. Lower panel reports odd ratios (ORs) of new-onset hypertension. Cups of coffee equal to zero is used as reference group. ORs are adjusted for age, sex, and relative blood pressure.

As illustrated in Fig. 2, the incidence (upper panel) and adjusted risk (lower panel) of new-onset office, home and 24 h ambulatory hypertension detected after 10-year follow-up were similar in nonconsumers and consumers, one to two and more than three cups of coffee per day.

**FIGURE 3:**
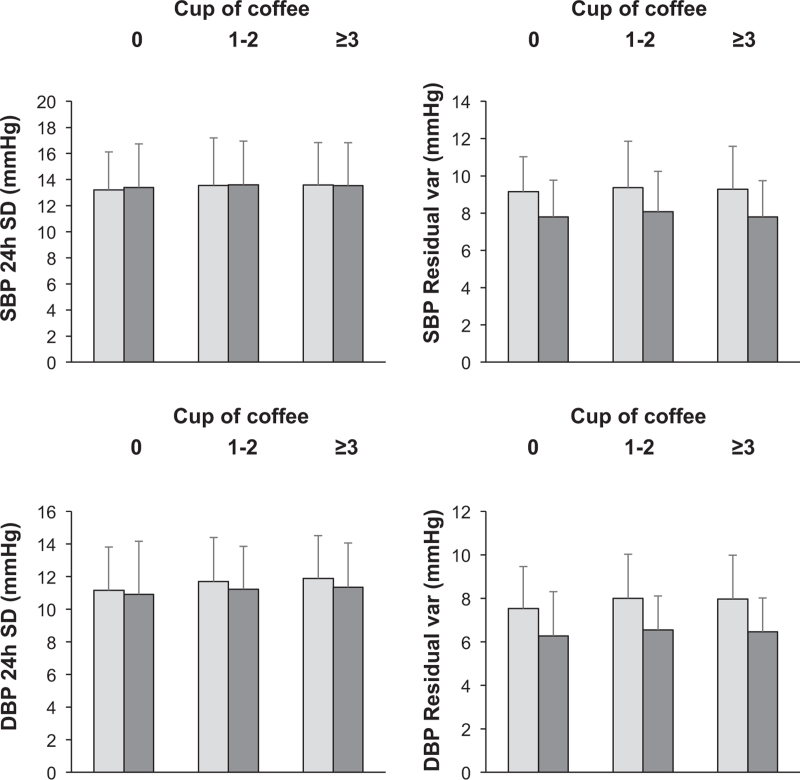
Left panels: twenty-four (24 h) SBP (upper panels) and DBP (lower panels) standard deviations in the three groups of subjects of Figure 1. Right panels: residual variability (Var) for SBP (Upper panels) and diastolic (DBP, lower panels) variables in the same groups of subjects. Data are shown as means ± standard deviation at the study entry (grey columns) and after 10 year follow-up (black columns).

Data related to the behaviour of different indices of BP variability in nonconsumers and consumers detected at the entry visit and after 10 years in the three groups of subjects of the present study are shown in Fig. 3, upper and lower panels referring to systolic and diastolic values, respectively. In all the three groups of subjects, SBP and DBP variability, expressed as 24 h standard deviation of the average values and as residual component, were, respectively, similar or slightly lower, when assessed after 10-year follow-up as compared with the values detected at the study entry. No significant difference was found between coffee nonconsumers and consumers.

Finally, data analysis based on the gender of the participants did not reveal any significant difference between men and women as far as the in-office and out-of-office blood pressure values and BP variability detected at the study entry and after 10 year follow-up in coffee nonconsumers and consumers (Supplemental Table 1) are concerned. This was also the case for heart rate, and for the of new-onset hypertension as well (data not shown).

## DISCUSSION

The present analysis of the data collected in the frame of the PAMELA study and related to the longitudinal long-term association between habitual coffee consumption and BP provides three novel results. First, it shows that office BP, both at the study entry and at the 10-year follow-up, was slightly but significantly lower in its systolic, but not diastolic, component in consumers who consume three or more cups of coffee per day than in nonconsumers or consumers who consume one to two cups daily. However, when home and 24 h values were taken into account, no significant difference in BP values both at the study entry and after 10 year follow-up was detected in coffee nonconsumers and consumers. This was the case when data were corrected for a number of confounders, such as age, BMI, antihypertensive drug treatment, HDL cholesterol, smoking and estimated glomerular filtration rate. Second, the study shows that during the prolonged follow-up also the occurrence of new hypertensive states, evaluated via office, home and 24 h ambulatory BP monitoring, was similar in coffee nonconsumers and consumers. Third, analysis of blood pressure variability data provides evidence that 24 h SBP and DBP variabilities, both when expressed as standard deviation of the average values and as residual variability, were virtually superimposable in coffee nonconsumers and consumers at the study entry and after 10 year follow-up.

Taken together, these three data sets allow to conclude that habitual coffee consumption is not associated with any significant long-term BP lowering or enhancing effect, the lower BP values detected via office measurements being of small magnitude, restricted to the systolic component only and, more importantly, not confirmed by home or 24 h measurements. It should be mentioned that the disagreement over the results obtained via office and out-of-office BP measurements detected in the present study is not peculiar to coffee consumption data but common to a number of other clinical conditions [21–22]. It may depend on several factors, such as the greater accuracy and reproducibility over time of out-of-office BP measurements as compared with the in-office ones [23]; the much greater number of measurements on which out-of-office (particularly 24 h ambulatory monitoring) BP evaluations are based as compared with the small number of in-office measurements [23]; and the presence of an alerting reaction to office, but not to out-of-office, BP measurements, which interferes with the correct assessment of the ‘true’ BP values [23–25].

In a recently published study based on a cross-sectional analysis of the first PAMELA study survey, we found that habitual coffee consumption does not appear to have any major lowering effect on BP values particularly when they are assessed via ambulatory or home BP monitoring [17]. The results of the present longitudinal study confirm and strengthen these findings, by providing prospective evidence that during the 10 year follow-up coffee consumption: neither reduces nor increases clinic, home and 24 h BP; does not affect the development of new hypertensive states evaluated not only via in-office but also via out-of-office BP measurements; and leaves unaltered BP variability.

Few other results of our study deserve to be briefly mentioned. First, both at the study entry and at the 10 year follow-up, no significant gender-related difference was found in the analysis of office, home, and 24 h BP values and their corresponding variabilities in coffee nonconsumers and consumers. Thus, our data rule out any possible gender-related difference in the association between coffee consumption and BP, as previously suggested [1,8,12]. Second, office, home, and 24 h heart rate values were similar in coffee nonconsumers and consumers both at the study entry and after 10 year follow-up, confirming prospectically the findings previously reported by our group in a cross-sectional study [17]. Third, changes in antihypertensive drug treatment occurring during the follow-up were homogeneous in the different coffee consumer groups and did not significantly affect the results. This was the case also for the different classes of antihypertensive drugs used.

The present study has strengths and limitations. The main strengths include the prospective nature of the present study, which is based on data collected during the longest follow-up never performed before in studies aimed at assessing the association between coffee consumption and BP. They also include the fact that assessment of BP values was based not only on clinic but also on home and 24 h measurements, thus providing information on relationships between coffee consumption, in-office, and out-of-office BP. The limitation is represented by the self-reported nature of coffee consumption, which may undergo inaccuracies in the finding. It should be emphasized, however, that this limitation is common to the majority of the published large-scale population studies on the association between coffee consumption and BP. A further limitation refers to the lack of information about possible modifications of specific nutrients and energy intake of the diet during the years. It should be mentioned, however, that no substantial dietary modification was reported by participants during the follow-up.

In conclusion, the results of the present longitudinal study conclusively document that habitual coffee consumption exerts neutral effects on in-office and out-of-office BP values and related variabilities. This is the case also for the new-onset hypertensive state.

## ACKNOWLEDGEMENTS

### Conflicts of interest

There are no conflicts of interest.

## Supplementary Material

Supplemental Digital Content
