# Supplementary material for: Habitual coffee consumption and office, home, and ambulatory blood pressure: results of a 10-year prospective study
Source: J Hypertens. 2024 Apr 22;42(6):1094–100. doi: 10.1097/HJH.0000000000003709 (PMC11064901; doi:10.1097/HJH.0000000000003709)
Supplement: Supplemental Digital Content [file jhype-42-1094-s001.docx]

**Supplemental Table 1.** Office, home and 24-hour blood pressure values and related blood pressure variability, expressed as standard deviation and residual cyclic component, in females and males.

|  | **Cups of coffee per day** | | | | | |
| --- | --- | --- | --- | --- | --- | --- |
|  | **0** | | **1-2** | | **≥3** | |
|  | **Baseline** | **10 year** | **Baseline** | **10 year** | **Baseline** | **10 year** |
| **Females, number** | **50** | | **245** | | **270** | |
| SBP Office, mmHg | 134.7±24 | 142.4±25.1 | 129.4±20.7 | 139.5±25.3 | 122.8±16.5 | 133.8±21.3 |
| DBP Office, mmHg | 82.2±10.1 | 82±8.6 | 81.8±10.2 | 83.5±10.8 | 79.5±9.6 | 82.4±10.8 |
| SBP Home, mmHg | 119±18.4 | 124.4±18.8 | 118.6±17.9 | 123.1±17.9 | 115.7±16.8 | 121.3±15.7 |
| DBP Home, mmHg | 72.4±10 | 71.4±8.7 | 74.4±10.5 | 72.2±8.6 | 72.7±10.1 | 72.8±8.6 |
| SBP 24h, mmHg | 117.1±9.9 | 123.1±12.3 | 117±10.9 | 121.8±11.8 | 115.2±10 | 120.4±10.9 |
| DBP 24h, mmHg | 71.5±6.6 | 72.3±7.4 | 72.4±6.4 | 72.3±6.3 | 71.5±6.4 | 72.3±7.4 |
| SBP 24h SD, mmHg | 12.8±2.3 | 13.7±3.4 | 13.3±3.5 | 13.4±3.3 | 13.2±3.4 | 13±3.1 |
| DBP 24h SD, mmHg | 10.8±2.6 | 10.8±3.6 | 11.6±2.7 | 11±2.5 | 11.7±2.8 | 10.8±2.6 |
| Residual Cyclic C SBP, mmHg | 9.2±1.8 | 8.1±2.2 | 9.3±2.4 | 8±2.1 | 9.2±2.5 | 7.5±1.7 |
| Residual Cyclic C DBP, mmHg | 7.4±1.9 | 6.3±2.4 | 8±2.1 | 6.5±1.5 | 7.9±2.2 | 6.2±1.4 |
|  |  |  |  |  |  |  |
| **Males, number** | **52** | | **226** | | **333** | |
| SBP Office, mmHg | 134.1±18.7 | 142.4±23.1 | 134.6±20.2 | 139.1±21.8 | 131.1±18.7 | 138.2±21.8 |
| DBP Office, mmHg | 85±9.8 | 85.4±10.4 | 86±10.6 | 84.2±10.6 | 85.3±9.8 | 84.9±11 |
| SBP Home, mmHg | 126.6±16.5 | 124.8±14.7 | 128.3±16.8 | 128.7±16 | 125.9±15 | 129.3±15.2 |
| DBP Home, mmHg | 77.9±9.8 | 73.7±8.1 | 78.1±10 | 76.2±8.5 | 77.7±9.3 | 78.1±8.6 |
| SBP 24h, mmHg | 120.5±11.8 | 123.3±12.2 | 122.7±11.5 | 125.7±11.9 | 122.4±10.5 | 126.7±11.7 |
| DBP 24h, mmHg | 74.4±7.2 | 73.4±8.1 | 77±7.7 | 76.3±7.7 | 76.8±6.9 | 78.1±8.1 |
| SBP 24h SD, mmHg | 13.6±3.4 | 13.1±3.3 | 13.8±3.8 | 13.8±3.4 | 13.9±3.1 | 14±3.4 |
| DBP 24h SD, mmHg | 11.5±2.7 | 11±2.9 | 11.8±2.7 | 11.5±2.7 | 12.1±2.4 | 11.8±2.8 |
| Residual Cyclic C SBP, mmHg | 9.1±1.9 | 7.5±1.7 | 9.4±2.6 | 8.2±2.2 | 9.4±2.2 | 8±2.1 |
| Residual Cyclic C DBP, mmHg | 7.7±2 | 6.2±1.6 | 8±2 | 6.7±1.6 | 8±1.8 | 6.7±1.6 |
|  | | | | | | |

Data are shown as means±standard deviations. SBP: systolic blood pressure, DBP:diastolic blood pressure; SD: standard deviation, C: component.
